# VMAT2-Mediated Neurotransmission from Midbrain Leptin Receptor Neurons in Feeding Regulation

**DOI:** 10.1523/ENEURO.0083-17.2017

**Published:** 2017-05-26

**Authors:** Yuanzhong Xu, Yungang Lu, Pingwen Xu, Leandra R. Mangieri, Elsa Isingrini, Yong Xu, Bruno Giros, Qingchun Tong

**Affiliations:** 1 Brown Foundation of Molecular Medicine for the Prevention of Human Diseases of McGovern Medical School, The University of Texas Health Science Center at Houston, Houston, TX, 77030; 2 Department of Psychiatry, Douglas Hospital, McGill University, Montreal, Quebec, Canada, H4H 1R3; 3 Children’s Nutrition Research Center, Department of Pediatrics, Baylor College of Medicine, Houston, TX, 77030; 4 Program of Neuroscience, MD Anderson and UTHealth Graduate School for Biological Sciences of the University of Texas Health Science Center at Houston, Houston, TX, 77030

**Keywords:** dopamine, HFD feeding, leptin, obesity, VMAT2

## Abstract

Leptin receptors (LepRs) expressed in the midbrain contribute to the action of leptin on feeding regulation. The midbrain neurons release a variety of neurotransmitters including dopamine (DA), glutamate and GABA. However, which neurotransmitter mediates midbrain leptin action on feeding remains unclear. Here, we showed that midbrain LepR neurons overlap with a subset of dopaminergic, GABAergic and glutamatergic neurons. Specific removal of vesicular monoamine transporter 2 (VMAT2) in midbrain LepR neurons (KO mice) disrupted DA accumulation in vesicles, but failed to cause a significant change in the evoked release of either glutamate or GABA to downstream neurons. While KO mice showed no differences on chow, they presented a reduced high-fat diet (HFD) intake and resisted to HFD-induced obesity. Specific activation of midbrain LepR neurons promoted VMAT2-dependent feeding on chow and HFD. When tested with an intermittent access to HFD where first 2.5-h HFD eating (binge-like) and 24-h HFD feeding were measured, KO mice exhibited more binge-like, but less 24-h HFD feeding. Interestingly, leptin inhibited 24-h HFD feeding in controls but not in KO mice. Thus, VMAT2-mediated neurotransmission from midbrain LepR neurons contributes to both binge-like eating and HFD feeding regulation.

## Significance Statement

Despite the well-established role for midbrain neurons in directly mediating leptin action on feeding, the identity of neurotransmitters that mediate the leptin action remains uncertain. Midbrain LepR neurons overlap with neurons that express vesicular transporters for dopamine [DA; vesicular monoamine transporter 2 (VMAT2)], for GABA [vesicular GABA transporter (VGAT)], or for glutamate [vesicular glutamate transporter 2 (VGLUT2)], posing a question on relative importance of these neurotransmitters in mediating leptin action. This study addressed the function of neurotransmitter release mediated by VMAT2 from ventral tegmental area (VTA) LepR neurons. Our data identified a role for VMAT2-mediated neurotransmission in both high-fat diet (HFD) feeding and binge-like eating behaviors, representing a significant step in understanding the midbrain leptin pathway in feeding regulation.

## Introduction

The current obesity epidemic, as a result of nutrient overconsumption, demands more mechanistic insights on feeding regulation related to nutrient overconsumption. Recently, increasing evidence demonstrates that nutrient overconsumption on high-fat, high-sucrose diet (HFD) feeding involves hedonic feeling, i.e., the drive to eat more than required food due to the pleasurable feelings associated with feeding (Saper et al., 2002; Kenny, 2011; Meye and Adan, 2014). Given the similarity in the drive for pleasure-seeking between hedonic feeding and drug abuse, the ventral tegmental area (VTA), a region extensively studied for drug abuse control, also plays an important role in hedonic feeding and nutrient overconsumption (Cordeira et al., 2010; Denis et al., 2015; Tellez et al., 2016).

A subset of VTA neurons express leptin receptors (LepRs), and sense changes in the level of leptin, a hormone released form adipose tissues and a key regulator for body weight and feeding (Fulton et al., 2006; Hommel et al., 2006). Knockdown of LepR in the VTA leads to higher food intake while activation of LepR by leptin reduces feeding (Hommel et al., 2006). In addition, deletion of Signal transducer and activator of transcription 3 (STAT3), a key signal molecule mediating leptin action, in the VTA leads to an increased level of locomotion related to reward (Fernandes et al., 2015). Leptin-deficient *ob/ob* mice exhibit an increased drive for feeding, reward, and hedonic feeding, suggesting a general role for leptin in suppressing hedonic feeding (Fulton et al., 2006).

The VTA is known to contain a mixture of dopaminergic, glutamatergic and GABAergic neurons. Importantly, recent data suggest that vesicular monoamine transporter 2 (VMAT2) in the midbrain also mediates presynaptic release of GABA, in addition to well-documented dopamine (DA) release (Tritsch et al., 2012; Kim et al., 2015). The availability of GABA appears not to depend on glutamate acid decarboxylase activity or vesicular GABA transporters (VGAT; Tritsch et al., 2014; Kim et al., 2015), which are otherwise a typical feature of GABAergic neurons. GABA release from DA neurons is important in mediating alcohol addiction and self-stimulation (Kim et al., 2015; Berrios et al., 2016), suggesting a previously underappreciated role for of other neurotransmitters coreleased with DA. In addition, a subset of VTA dopaminergic neurons also express vesicular glutamate transporter 2 (VGLUT2) and are capable of releasing glutamate (Hnasko et al., 2012). VTA LepR neurons partially overlap with dopaminergic and GABAergic neurons (Fulton et al., 2006). However, the identity of neurotransmitters that mediate leptin action in the VTA is unknown.

Here, we demonstrated that among all brain LepR neurons, only those in the midbrain express VMAT2. In addition, a subset of VTA LepR neurons also express VGAT and VGLUT2. To identify the role of VMAT2-mediated neurotransmission in mediating leptin action, we generated mice with specific deletion of VMAT2 in LepR neurons. Our data showed that deletion of VMAT2 deletion resulted in disrupted accumulation of DA in vesicles and reduced HFD feeding, but increased binge-like eating.

## Materials and Methods

### Animals

Mice were housed at 21-22°C with a 12/12 h light/dark cycle with food and water provided *ad libitum*. Animal care and procedures were approved by the Animal Welfare Committee of our university. *LepR-Ires-Cre* (*LIC*) and *Vmat2^fl^°^x/fl^°^x^*mice were described previously (Narboux-Neme et al., 2011; Xu et al., 2013; Isingrini et al., 2016). Breeding pairs (male *LIC:Vmat2^fl^°^x/+^* mice and female *Vmat2^fl^°^x/+^*mice) were maintained to generate the study subjects. Male and female *Vmat2^fl^°^x/+^* mice were interbred to generate wild-type and *Vmat2^fl^°^x/fl^°^x^* mice for AAV-Cre injection studies described below. In addition, *LIC* mice were bred to Ai9 reporter mice (Madisen et al., 2010) to generate *LIC::Ai9* mice for electrophysiological recording or colocalization between LepR expression and VMAT2 or tyrosine hydroxylase (TH). *Vgat-Ires-Cre* and *Vglut2-Ires-Cre* mice were described previously (Vong et al., 2011) and purchased from The Jackson Laboratory and were stereotaxically injected with the adeno-associated viral vector AAV-FLEX-GFP, which express GFP in a Cre-dependent manner, to visualize GABAergic or glutamatergic neurons, respectively, in the midbrain.

### Studies with stereotaxic injections

After deep anesthesia with ketamine and xylazine, mice were placed on a stereotaxic frame (David Kopf Instruments). Adeno-associated viral vectors, AAV-GFP-Cre, AAV-GFP, AAV-DIO-hM3D(Gq)-mCherry (i.e., designer receptor exclusively activated by designer drugs, DREADD), AAV-FLEX-GFP and AAV-FLEX-ChR2-YFP vectors were purchased from the viral core facility of the University of Pennsylvania or University of North Carolina and were stereotaxically injected into bilateral VTA (400 nl each site) with the following coordinates: bregma, +3.1 mm; midline, ±0.4 mm; dorsal surface, −5.0 mm, using a 0.5-µl Hamilton syringe controlled by a nano-injector (Braintree). The injection speed was 50 nl/min, and the syringe was withdrawn 5 min after the final injection. For intracerebraventricular (i.c.v.) cannulation, the following coordinates were used: bregma, +0.3 mm; midline, ±1.0 mm; dorsal surface, −2.5 mm. Mice were used for experiments after a four-week period recovery.

### Body weight and food intake measurements

Weekly body weight and food intake were monitored in all genotypes fed standard mouse chow (Teklad F6 Rodent Diet 8664, 4.05 kcal/g, 3.3 kcal/g metabolizable energy, 12.5% kcal from fat, Harlan Teklad) or HFD (D12331, 58% kcal from fat Research Diet) from four-week-old mice.

For binge-like eating pattern measurements, we used a previously reported protocol (Czyzyk et al., 2010; Cao et al., 2014). Briefly, mice were randomly assigned into an intermittent or continuous group. Mice in the intermittent group were exposed to both regular chow pellets and HFD for 24 h and then exposed to only chow for the rest of the week, which composes a cycle. On the test day, HFD was given back to cages, and HFD and chow intake were measured for both 2.5 and 24 h after HFD diet was placed. The continuous group was exposed to chow and HFD for the entire study, and HFD and chow intake were similarly measured for both 2.5 and 24 h as in the intermittent group. Mice were singly housed for the entire study period and the measurements were repeated for six cycles. Previous studies using this paradigm suggest that mice in the intermittent group consumed a large amount of HFD during the initial 2.5 h on the test day, which was considered to be binge-like eating behavior (Czyzyk et al., 2010; Cao et al., 2014). To test the effect of leptin on feeding in this paradigm, mice were intraperitoneal treated with murine recombinant leptin (A.F. Parlaw, National Hormone and Peptide Program) twice during the measurement cycle (2 μg/g body weight) before the addition of HFD during testing periods.

For food intake measurements in mice with acute midbrain LepR neurons activation, AAV-DIO-hM3D(Gq)-mCherry vector-injected mice were intraperitoneally injected with clozapine N-oxide (CNO, 1 mg/kg) or saline, food intake recorded 30 min, 1 h, 2 h, and 4 h after CNO administration was compared between genotypes. Food intake studies were conducted four weeks after viral delivery, and saline and CNO were administered in a crossover fashion with one-week interval.

### Immunohistochemistry assays

For immunohistochemistry studies, primary antibodies against VMAT2 (Phoenix Pharmaceuticals), TH (Abcam), or p-STAT3 (Phoenix) were used. For the DA immunohistochemistry study, *LIC::Ai9* mice were perfused and the brain sections were prepared according to the manufacturer protocol for DA detection (MAB5300, EMD Millipore). All brain sections were incubated with the primary antibodies overnight at room temperature following 1 h blocking in 10% normal donkey serum. After visualized with secondary donkey IgG serum conjugated with Alexa Fluor 488, Alexa Fluor 574, or Alexa Fluor 647 (Jackson ImmunoResearch), sections were photographed with a TCS SP5 confocal microscope (Leica).

### Brain slice electrophysiological recordings

Horizontal slices (220 μm) containing the ventral midbrain from *LIC:Ai9* (for recording leptin effects on LepR neurons), or from *LIC::Vmat2^fl^°^x/fl^°^x^* mice stereotaxically injections of AAV-Cre-eGFP or AAV-eGFP (for recording DA-mediated currents) to the VTA, were cut in ice-cold artificial cerebrospinal fluid (aCSF) containing the following: 125 mM NaCl, 2.5 mM KCl, 1 mM MgCl_2_, 2 mM CaCl_2_, 1.25 mM NaH_2_PO_4_, 25 mM NaHCO_3_, and 11 mM D-glucose bubbling with 95% O_2_/5% CO_2_. Slices containing the VTA were immediately transferred to a holding chamber and submerged in oxygenated aCSF. Slices were maintained for recovery for at least 1 h at 32 -34°C before transferring to a recording chamber. Individual slices were transferred to a recording chamber mounted on an upright microscope (Olympus BX51WI) and continuously superfused (2 ml/min) with aCSF warmed to 32-34°C by passing it through a feedback-controlled in-line heater (TC-324B; Warner Instruments). Cells were visualized through a 40× water-immersion objective with either infrared differential interference contrast optics or epifluorescence to identify tdTomato positive LepR-expressing neurons. Physiologic identification was based on the presence of spontaneous pacemaker firing at 1–5 Hz.

To record the effect of leptin on the firing property of action potential of VTA LepR neurons, pipettes for whole-cell patch clamp (2–3 MΩ) were filled with a solution containing 145 mM K-gluconate, 1 mM MgCl_2_, 10 mM HEPES, 0.2 EGTA, 4 mM ATP, 0.3 mM GTP, and 10 mM phosphocreatine, pH 7.35, 270–285 mOsm, with 0.2% biocytin.

For channelrhodopsin 2 assisted circuitry mapping (CRACM), using a similar procedure to that for VTA slices, coronal brain slices (250-300 µm) containing the nucleus accumbens or amygdala were prepared from mice that had received injections of AAV-FLEX-ChR2-YFP to bilateral VTA at least four weeks before the recording. Whole cell voltage-clamp recordings were made from neurons within the subregions of the nucleus accumbens or amygdala that showed the highest density of ChR2-YFP+ axonal fibers. Patch pipettes (2–3 MΩ) pulled from borosilicate glass (BF150-110, Sutter Instruments) were filled with a Cs+-based low Cl^−^ internal solution containing 135 mM CsMeSO_3_, 10 mM HEPES, 1 mM EGTA, 3.3 mM QX-314, 4 mM Mg-ATP, 0.3 mM Na-GTP, 8 mM Na2-phosphocreatine, pH 7.3 adjusted with CsOH; 295 mOsm; for voltage-clamp recordings. Membrane potentials were corrected for ∼10-mV liquid junction potential. To activate ChR2-expressing fibers, light from a 473 nm laser (Opto Engine LLC) was focused on the area of the recorded neuron to produce spot illumination on the brain section. Brief pulses of light (1-ms flash of blue light; 1-2 mW/mm^2^) were delivered at the recording site at 15 s intervals under control of the acquisition software. TTX was from Alomone labs, and 4-AP from ACROS Organics (Fisher Scientific).

### Statistical analysis

Data are expressed wherever possible as mean ± SEM. All analytical statistics were performed with GraphPad Prism (GraphPad Software). Two groups were made with unpaired Student’s *t* tests, and statistical significance was defined as *p* < 0.05. Multiple comparison analyses were performed by using one- or two-way analysis of variances followed by Tukey *post hoc* tests.

## Results

### VMAT2 expression in LepR neurons

**Figure 1.**
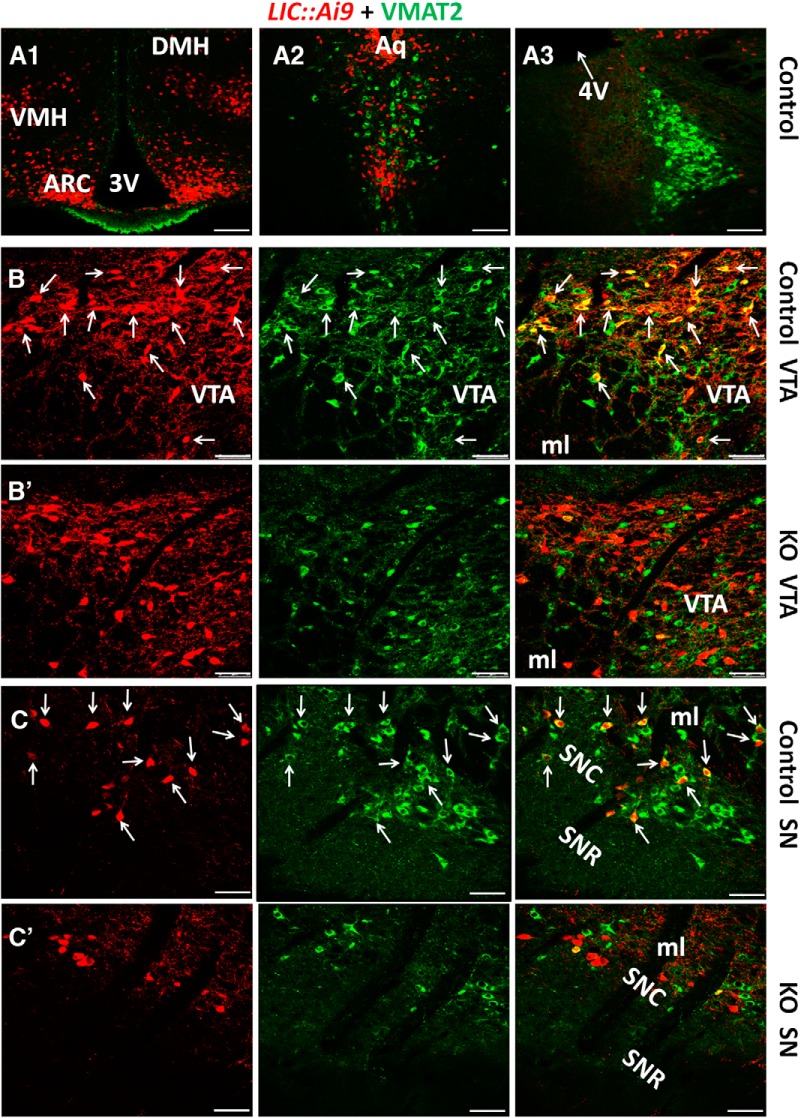
Selective deletion of VMAT2 in midbrain LepR neurons. Immunostaining of VMAT2 was performed on brain slices of *LIC:Ai9* reporter mice, which express tdTomato (red) in a Cre-dependent manner. No colocalization of VMAT2 (green) and tdTomato (red) was found in the hypothalamus (***A1***), dorsal raphei (***A2***), or locus coeruleus (***A3***). LepR (***B***, ***C***, left panels) and VMAT2 expression (***B***, ***C***, middle panels) were found in the VTA (***B***) and SN (***C***) of control mice. VMAT2 was found in a subset of LepR neurons in the VTA (***B***, right panel, arrows) and in SN (***C***, right panel, arrows). LepR (***B’***, ***C’***, left panels) and VMAT2 (***B’***, ***C’***, middle panels) were also found in the VTA (***B’***) and SN (***C’***) of *LIC::Vmat2^fl^°^x/fl^°^x^* mice. VMAT2 expression was undetectable in LepR neurons in VTA (***B’***, right panel) or SN (***C’***, right panel) of *LIC::Vmat2^fl^°^x/fl^°^x^* mice. ARC, arcuate nucleus; DMH, dorsomedial hypothalamus; VMH, ventromedial hypothalamus; ml, medial lemniscus; SNC, SN compact; SNR, SN retic part; 3V, third ventricle; 4V, fourth ventricle. Scale bars, 100 μm (***A1–A3***) and 50 μm (***B***, ***B’***, ***C***, ***C’***).

To examine potential colocalization between LepR and VMAT2, we immunostained VMAT2 expression in brain slices of *LIC::Ai9* reporter mice. VMAT2 expression was not found in LepR neurons in the hypothalamus (Fig. 1*A1*). Although it was found in the dorsal raphei (Fig. 1*A2*) and the locus coeruleus nucleus (Fig. 1*A3*), VMAT2 was not colocalized with LepR in either site (Fig. 1*A2*,*A3*). LepR (Fig. 1*B*,*C*, left panels) and VMAT2 (Fig. 1*B*,*C*, middle panels) were found to be colocalized in a subset of midbrain neurons including those located in both VTA (Fig. 1*B*, right panel) and substantial nigra (Fig.1*C*, right panel). Notably, a subset of LepR neurons in the VTA were VMAT2-negative (arrow heads; Fig. 1*B*, right panel). Cell counting from five male mice showed that around 68% of midbrain LepR neurons were dopaminergic, and ∼ 28% of midbrain dopaminergic neurons showed LepR expression. LepR (Fig. 1*B’*,*C’*, left panels) and VMAT2 (Fig. 1*B’*,*C’*, middle panels) were found to be colocalized in neither VTA (Fig. 1*B’*, right panel) nor substantia nigra (SN) neurons (Fig. 1*C’*, right panel) of *LIC::Vmat2^fl^°^x/fl^°^x^* mice (KO). In addition, VMAT2 was not found in LepR neurons in all other brain sites examined (data not shown). These data suggest that only midbrain LepR neurons express VMAT2, and in KO mice, VMAT2 was specifically deleted in midbrain LepR neurons.

**Figure 2.**
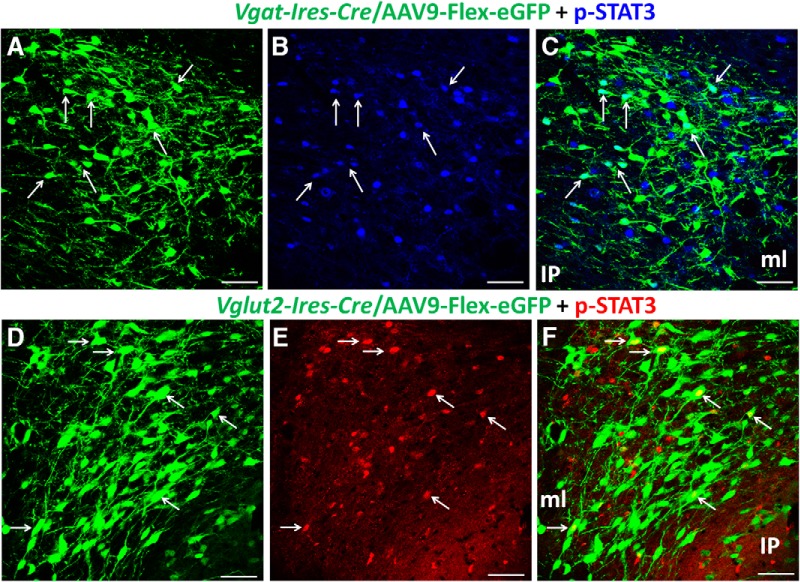
A subset of VTA LepR neurons are glutamatergic or GABAergic. Immunostaining of pSTAT3 was performed on brain sections of mice that received i.c.v. injection of leptin. These mice also received stereotaxic injections to the VTA of AAV-FLEX-GFP vectors to allow labeling of Cre-expressing neurons with GFP. Expression of GFP (***A***, ***D***) and p-STAT3 (***B***, ***E***) was found in the VTA of *Vgat-Ires-Cre* mice (***A–C***) and *Vglut2-IRES-Cre* mice (***D–F***). p-STAT3 positive neurons were found in a subset of GFP-expressing neurons in both *Vgat-Ires-Cre* mice (***C***, arrows) and *Vglut2-IRES-Cre* mice (***F***, arrows). IP, interpeduncular nucleus; ml, medial lemniscus. Scale bars, 25 μm.

Since we observed that a subset of midbrain VTA LepR neurons were VMAT2 negative, we explored whether these neurons were glutamatergic or GABAergic. To identify LepR neurons, we used p-STAT3 as a surrogate marker, which can be induced by i.c.v. administration of leptin. Due to unknown reasons, Cre-mediated expression of tdTomato in either *Vglut2-Ires-Cre::Ai9* or *Vgat-Ires-Cre::Ai9* reporter mice failed to label distinct neuron morphology, which is required to assess colocalization. We thus labeled Cre-expressing neurons in *Vglut2-Ires-Cre* and *Vgat-Ires-Cre* mice by stereotaxic injection to the VTA of AAV-FLEX-GFP vectors, which express GFP in a Cre-dependent manner. p-STAT3 (Fig. 2*A*,*D*) and GFP (Fig. 2*B*,*E*) were colocalized in a subset of VTA neurons of both *Vgat-Ires-Cre* (Fig. 2*C*) and *Vglut2-Ires-Cre* mice (Fig. 2*F*). Given the nature of potential incomplete targeting of Cre-expressing neurons with stereotaxic injections of Cre-dependent AAV vectors, this method may underestimate the number of *Vgat*- and *Vglut2*-expressing neurons in the VTA. In addition, because of i.c.v. leptin administration, a possibility for p-STAT3 induction by network effects induced by leptin action in non-VTA areas cannot be ruled out. Nonetheless, these results suggest that a subset of LepR neurons in the midbrain use VGAT or VGLUT2-mediated release of GABA or glutamate.

### Deletion of VMAT2 led to defective accumulation of DA in vesicles

**Figure 3.**
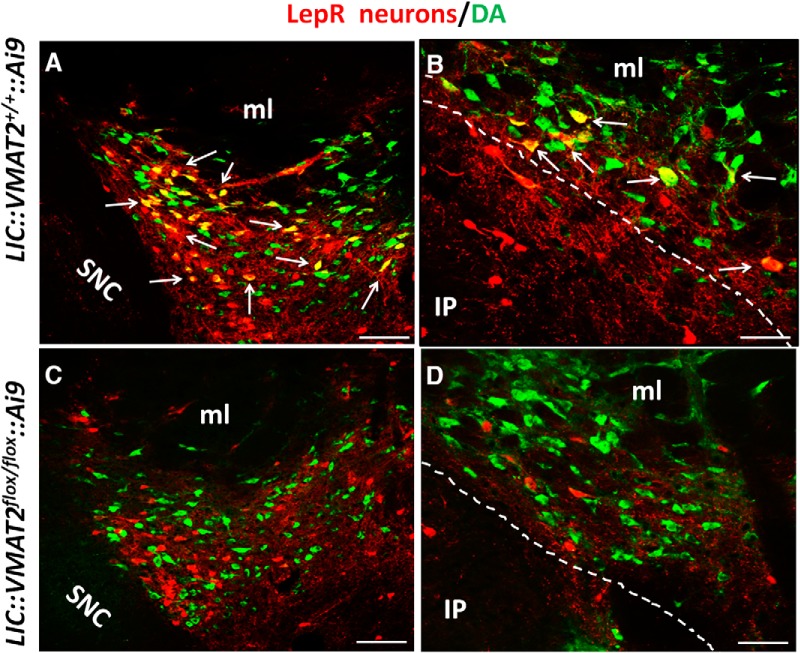
DA accumulation in vesicles was specifically disrupted from midbrain LepR neurons in *LIC::Vmat2^fl^°^x/fl^°^x^*mice. In the VTA, majority of LepR neurons (red) were costained by DA (green) in *LIC::VMAT2^+/+^::Ai9* mice (***A***, ***C***). However, no DA immunoreactivity was observed in VTA LepR neurons of *LIC::VMAT^fl^°^x/fl^°^x^::Ai9* mice (***B–D***). Arrows pointed neurons with strong DA immunoreactivity. IP, interpeduncular nucleus; ml, medial lemniscus; SNC, SN compact. Scale bars, 100 μm (***A***, ***C***) and 25 μm (***B***, ***D***).

Double immunostaining for VMAT2 and DA revealed that both immunoreactivity is abundant in neuron soma (Fig. 3*A*,*B*), which may reflect accumulation of a large number of vesicles. While a subset of LepR neurons were positive for DA (Fig. 3*A*,*B*, arrows), none was positive for DA in KO mice (Fig. 3*C*,*D*). Although a direct DA measurement in vesicles is required to confirm defective DA accumulation, the contrasting immunostaining results between controls and KOs suggest that deletion VMAT2 leads to disrupted accumulation of DA in vesicles, and therefore defective synaptic release of DA.

### Leptin inhibits VTA LepR neurons

**Figure 4.**
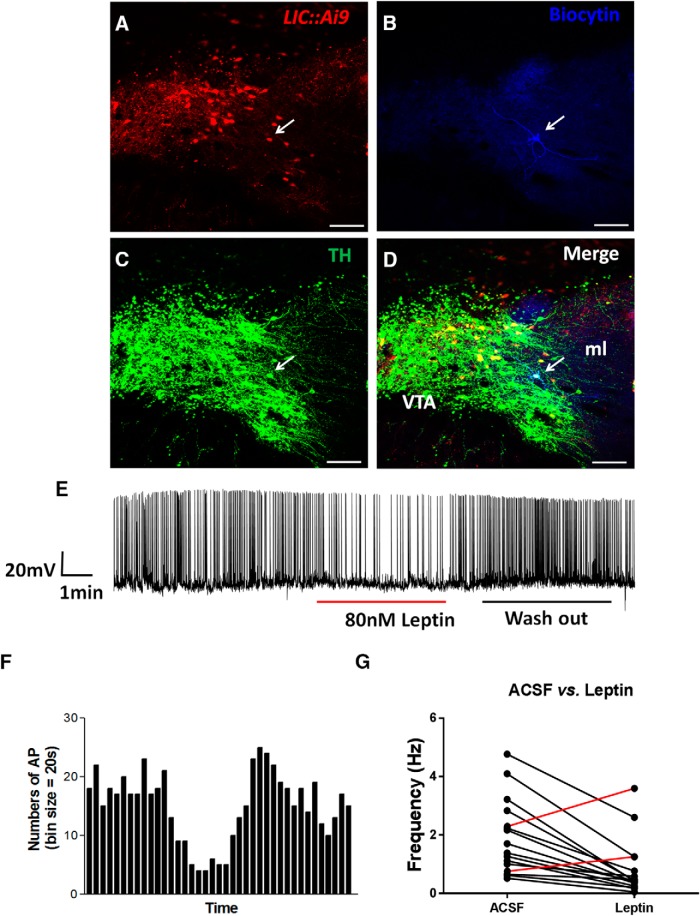
Leptin inhibits midbrain LepR neurons. Electrophysiological recording were performed on LepR neurons identified by tdTomato (red) expression in VTA brain sections of *LIC::Ai9* mice. In total, 16 out of 34 neurons responded to leptin. Representative images showing VTA brain sections with one LepR neuron identified for recording (***A***, arrow), immunostaining of injected biocytin during recording (***B***, blue), immunostaining of TH (***C***, green) and the merged (***D***). A representative recording trace showing changes in actual firing frequency (***E***) and the histogram of firing frequency (***F***) in response to leptin and washout. ***G***, Summary of firing frequency changes in response to leptin of all neurons that responded to leptin. Scale bars, 100 μm.

To examine the response of VTA dopaminergic LepR neurons to leptin, we recorded these neurons with tdTomato expresson (Fig. 4*A*) in brain slices of *LIC::Ai9* reporter mice. To ensure that the recorded neurons were DA neurons, we injected biocytin (Fig. 4*B*) during the recording through recording pipettes and performed *post hoc* immunostaining with TH antibody (Fig. 4*C*,*D*). The firing frequency of the majority of neurons recorded with response to leptin was reversibly reduced by leptin (Fig. 4*E*,*F*). Out of total 34 TH neurons recorded, 14 were inhibited by leptin, and 2 were slightly activated by leptin (Fig. 4*G*), and the remaining showed no significant responses. These results suggest that the overall effect of leptin on VTA dopaminergic LepR neurons is inhibitory.

### The effect of VMAT2 deletion on GABA and glutamate release

**Figure 5.**
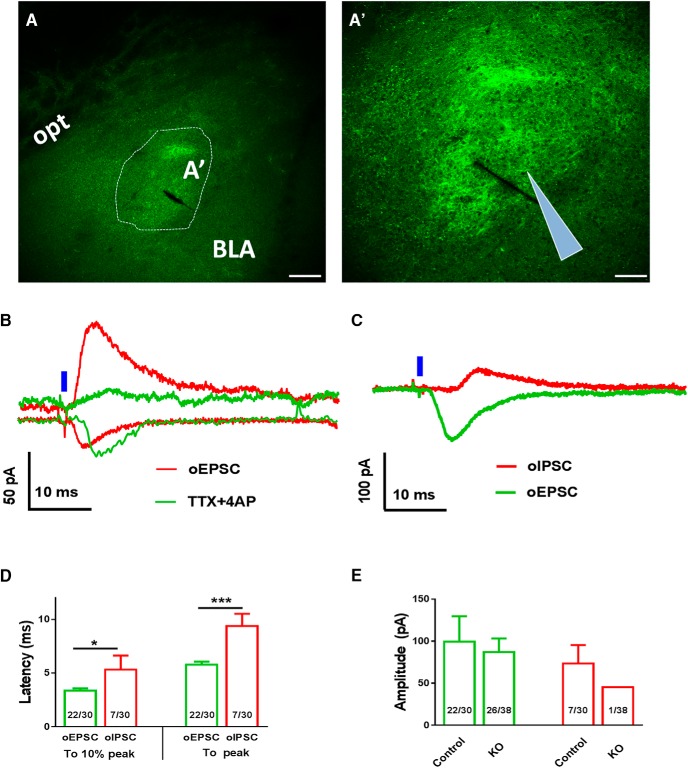
Effects of VMAT2 deletion in VTA LepR neurons on synaptic neurotransmitter release to the amygdala. Recordings of oEPCSc and oIPSCs were made in amydalar neurons elicited by blue laser stimulation of ChR2-expressing fibers from VTA LepR neurons in brain slices. ***A–A’***, Representative pictures showing ChR2-expressing fibers (green) in the central amygdala in low (***A***) and high magnification (***A’***). Representative recording traces of light evoked oEPSCs and oIPSCs from brain slices of control (***B***) and *LIC::Vmat2^fl^°^x/fl^°^x^* (KO) mice (***C***). Responses of oEPSCs and oIPSCs to TTX/4-AP were also shown in controls (***B***). ***D***, Quantitative analyses illustrate the latency for light-evoked oIPSCs (red) and oEPSCs (green) to reach 10% peak and 100% peak of the evoked currents from the onset of laser stimulation. ***E***, Mean amplitudes of oIPSC (red) and oEPSC (green) in controls and *LIC::Vmat2^fl^°^x/fl^°^x^* mice. Numbers in columns ***D***, ***E*** showed frequency of successful detection of indicated currents in all recorded neurons. BLA, basolateral amygdalar nucleus; opt, optic tract. Scale bars, 100 μm (***A***) and 50 μm (***A’***). Data were presented as mean ± SEM; *p < 0.05, ***p < 0.001, unpaired Student’s *t* tests.

To determine if deletion of VMAT2 in LepR neurons leads to loss of GABA or glutamate release, we used ChR2-assisted circuit mapping (CRACM). We stereotaxically delivered AAV-FLEX-ChR2-YFP to bilateral VTA of both *LIC* and KO mice. Four weeks after the delivery, we observed numerous ChR2-expressing fibers in the amygdala and a few of these fibers in the VTA. (Fig. 5*A*,*A’*), which is consistent with the previous result that VTA LepR neurons preferentially project to the amygdala (Leshan et al., 2010). We then recorded both EPSCs (oEPSCs) and IPSCs (oIPSCs) elicited by blue laser (473 nm) stimulation from randomly selected neurons in the amygdalar regions with abundant ChR2-expressing fibers. In *LIC* mice, we detected both oEPSCs and oIPSCs (Fig. 5*B*). While oEPSCs were not sensitive to TTX and 4-AP, which is routinely used to eliminate effects from multisynaptic neurotransmission, oIPSCs were eliminated by these drugs (Fig. 5*B*). Interestingly, in KO mice, we also successfully recorded both oIPSCs and oEPSCs (Fig. 5*C*). Notably, the latency to reach peak or 10% of peak of these currents after the onset of laser illumination was significantly longer for oIPSCs, compared with oEPSCs (Fig. 5*D*). These data suggest that the recorded oEPSCs were mediated by monosynaptic neurotransmission while oIPSCs were mediated through multisyanptic neurotransmission. The amplitude and ratio of recorded oEPSCs were not different between controls and KO mice (Fig. 5*E*), suggesting that glutamate release from LepR neurons were not affected by VMAT2 deletion. Interestingly, the frequency of successful detection of oIPSCs was very small in KO mice (1 out of 38), which might be a secondary effect to loss of DA release. Changes in DA release might affect local GABAergic neurotransmission, as shown in the lateral septum for changes in glutamate release (Chee et al., 2015).

**Figure 6.**
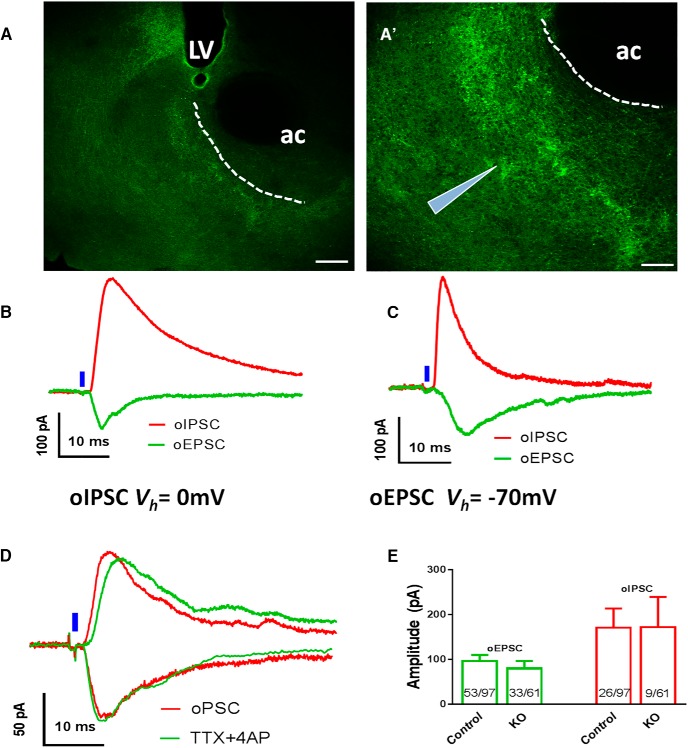
Effects of VMAT2 deletion in VTA LepR neurons on synaptic neurotransmitter release to the accumbens. Recordings of oEPCSc and oIPSCs were made in accumbens neurons elicited by blue laser stimulation of ChR2-expressing fibers from VTA LepR neurons in brain slices. Representative pictures showing ChR2-expressing fibers in the accumbens in low (***A***) and high magnification (***A’***). Representative recording traces of light evoked oEPSCs and oIPSCs from brain slices of control (***B***) and *LIC::Vmat2^fl^°^x/fl^°^x^* (KO) mice (***C***). ***D***, Representative traces showing responses of light-evoked oEPSCs and oIPSCs to TTX/4-AP in controls. ***E***, Mean amplitudes of light evoked oIPSC (red) and oEPSC (green) in controls and *LIC::Vmat2^fl^°^x/fl^°^x^* mice; numbers in the bar showed frequency of successful detection of the indicated currents in recorded neurons. Data were presented as mean ± SEM; ac, anterior commissure; LV, lateral ventricle. Scale bars, 100 μm (***A***) and 50 μm (***A’***).

We also found ChR2-expressing fibers in the accumbens (Fig. 6*A*,*A’*), suggesting a direction projection from VTA LepR neurons. Both oEPSCs and oIPSCs were elicited in accumbens neurons by blue laser in both control *Leptin-Ires-Cre* mice (Fig. 6*B*) and KO mice (Fig. 6*C*). Notably, both oEPSCs and oIPSCs were resistant to TTX/4-AP (Fig. 6*D*), suggesting monosynaptic connectivity. The amplitude of neither oEPSCs nor oIPSCs was different between *LIC* and KO mice (Fig. 6*E*). Furthermore, the frequency of successful detection of oEPSCs (53/97 vs 33/61) and oIPSCs (26/97 vs 9/61) recorded were largely comparable between genotypes. Thus, VMAT2 deletion in VTA LepR neurons failed to cause a significant difference in release of glutamate to amygdalar neurons, and release of GABA or glutamate to accumbens neurons.

### VMAT2 deletion in LepR neurons protected diet-induced obesity

**Figure 7.**
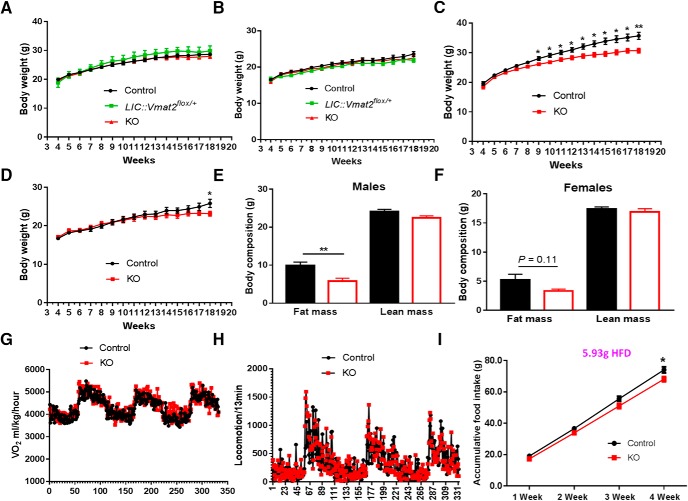
VMAT2 deletion in midbrain LepR neurons led to resistance to diet-induced obesity. Littermate mice were fed chow or HFD after weaning and weekly body weight and food intake were measured. Weekly body weight was measured in mice fed regular chow in males (***A***, *n* = 5–12) and females (***B***, *n* = 5–11), or HFD in males (***C***, *n* = 13–25) and females (***D***, *n* = 12–16). ***E***, ***F***, Body composition measurements in males (***E***, *n* = 12) and females (***F***, *n* = 12) fed with HFD at 18 weeks old. ***G***, ***H***, Energy expenditure assessed by O_2_ consumption (***G***, *n* = 6) and locomotion assessed by beam breaks (***H***, *n* = 6) measured by CLAMS in control and *LIC::Vmat2^fl^°^x/fl^°^x^* mice fed HFD diet at the age of 8–10 weeks. ***I***, Measurements of accumulated HFD food intake at ages of 8–12 weeks (*n* = 6–7). Data were presented as mean ± SEM; *p < 0.05, **p < 0.005 versus control mice, unpaired Student’s *t* tests on each measurement time point.

When fed chow, KO and control mice showed no difference in body weight in both males and females (Fig. 7*A*,*B*), and consistently, there was no difference in feeding and energy expenditure between genotypes (data not shown). When challenged with HFD feeding, KO mice showed a significantly lower body weight in both males (Fig. 7*C*) and females (Fig. 7*D*), although the degree in body weight difference was smaller in females compared with males. Notably, a smaller difference in females is not caused by differential expression of VMAT2 in females since 65% VTA LepR neurons in females were found to express TH and 30% of TH neurons were found to express LepR (data not shown), both in a similar range to males. Compared with controls, KO mice fed 15-week HFD exhibited significantly less fat mass in males (Fig. 7*E*) and a trend for lower fat mass in females (Fig. 7*F*), while there was no difference in lean mass (Fig. 7*E*,*F*), suggesting a lean phenotype. The reduced fat growth was not due to increased energy expenditure as O_2_ consumption measured on HFD showed no difference between genotypes (Fig. 7*G*). There was no difference in locomotion between genotypes in either day or dark periods measured by beam breaks (Fig. 7*H*) or E-mitters (data not shown). KO mice showed significantly lower HFD intake, compared with controls, during a period of four weeks (Fig. 7*I*). These results suggest that loss of VMAT2-mediated neurotransmission from LepR neurons reduces HFD feeding and protect diet-induced obesity.

### Acute stimulation of VTA LepR neurons increased feeding

**Figure 8.**
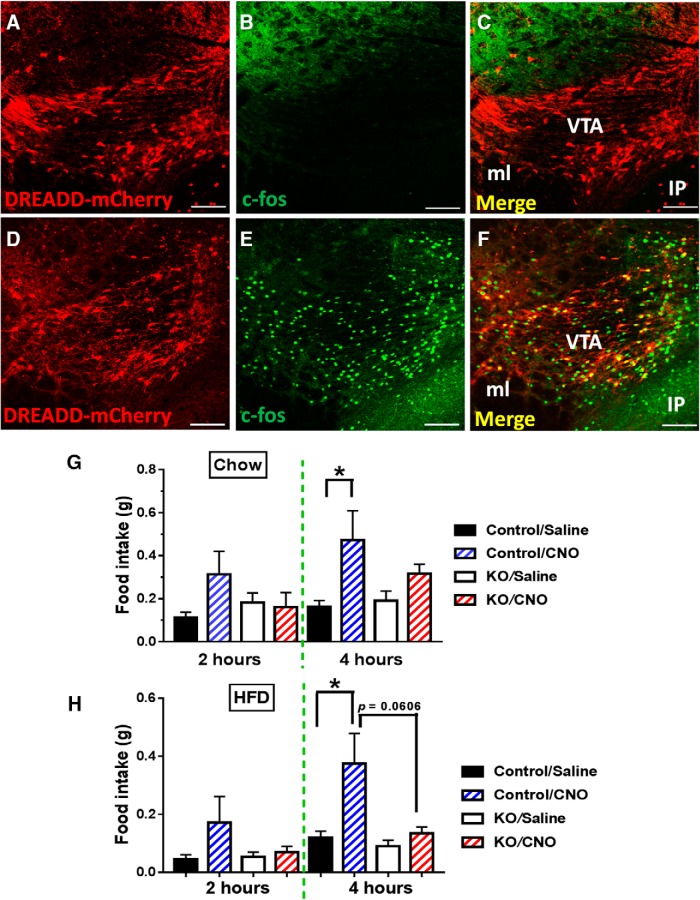
Acute activation of midbrain LepR neurons increased food intake. *LepR-Ires-Cre* and KO mice received stereotaxic injections of AAV-DIO-hM3D(Gq)-mCherry vectors to the VTA and food intake was measured four weeks after the injection. ***A–C***, Expression pattern of AAV-DIO-h3MD(Gq)-mCherry vectors (***A***), c-Fos (***B***), and their colocalization (***C***) in the VTA of *LepR-Ires-Cre* mice treated with saline. ***D–F***, Expression pattern of AAV-DIO-hM3D(Gq)-mCherry vectors (***D***), c-Fos (***D***), and their colocalization (***F***, arrows) in the VTA of *LepR-Ires-Cre* mice treated with CNO. ***G***, Food intake measured in early morning during the indicated time periods from mice fed chow treated with saline or CNO. ***H***, Food intake measured in early morning during the indicated time periods from mice fed HFD treated with saline or CNO. Scale bars, 50 μm; data were presented as mean ± SEM; *n* = 5–6 (***F***, ***G***), two-way ANOVA tests.

To further investigate the role of VTA LepR neurons in feeding regulation, we aimed to specifically activate this group of neurons using the DREADD approach. Toward this, we expressed AAV-DIO-hM3D(Gq)-mCherry vectors in VTA LepR neurons through bilateral stereotaxic delivery of these vectors to the VTA (Fig. 8*A*,*D*). We then used c-Fos as a marker to confirm activation of LepR neurons by CNO. While saline treated mice showed negligible c-Fos expression in LepR neurons (Fig. 8*B*,*C*), CNO produced a large number of c-Fos positive LepR neurons (Fig. 8*E*,*F*), confirming functional expression of DREADD receptors in VTA LepR neurons. While control mice treated with CNO showed a trend for increased food intake on chow (Fig. 8*G*), they showed an almost 3-fold increase feeding on HFD (Fig. 8*H*), suggesting a preference for HFD feeding on activation of VTA LepR neurons. In contrast, KO mice showed no response to CNO treatment. These results demonstrate that VMAT2-mediated neurotransmission mediates the effect of acute activation of VTA LepR neurons in promoting feeding.

### Binge-like eating and leptin-sensitive HFD feeding

**Figure 9.**
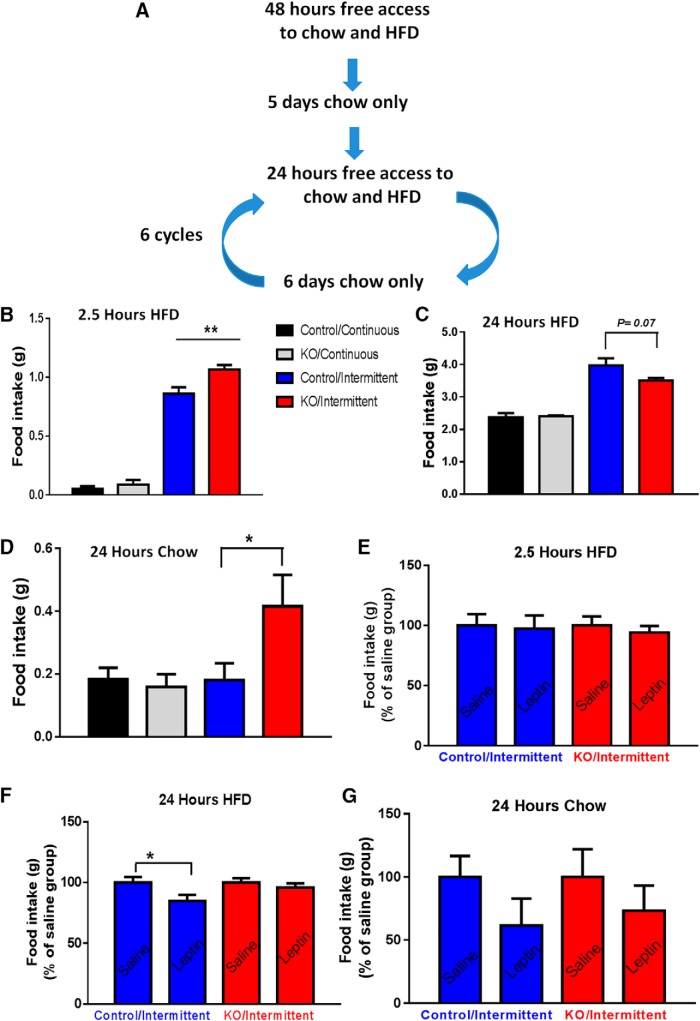
Selective VMAT2 deletion in midbrain LepR neurons led to increased binge-like eating. HFD food intake (2.5 and 24 h) was measured an intermittent HFD access setting in control and *LIC::Vmat2^fl^°^x/fl^°^x^* mice. ***A***, Diagram showing one cycle of total six cycles of the protocol used to measure binge-like eating (2.5-h feeding) and hedonic feeding (24-h feeding). A control experiment using a protocol with the presence of both chow and HFD all time was conducted in parallel. ***B–D***, Food intake was measured during initial 2.5-h HFD feeding (***B***), 24-h HFD feeding (***C***), and 24-h chow feeding (***D***) on the test day with HFD added. Initial 2.5-h chow feeding during the test day was not presented due to the negligible amount of food consumed. Response to leptin of initial 2.5-h HFD feeding (***E***), 24-h HFD feeding (***F***), and 24-h chow feeding (***G***) on the test day with HFD added. Data were presented as mean ± SEM, and each subject data were the average of food intake over cycles 3–5. *p < 0.05, *n* = 7–8, two-way ANOVA tests with Tukey *post hoc* analyses.

Previously, an intermittent HFD testing paradigm was developed to assess binge eating behavior (Czyzyk et al., 2010; Cao et al., 2014). In this paradigm, control groups receive a mixture of chow and HFD *ad libitum* for the whole duration of study while the experimental group, over every 7 d period, will only receive a mixture of chow and HFD for one day period (testing day) and receive chow diet only during the rest 6 days (Fig. 9*A*). The mice that receive intermittent HFD access consume a large amount of HFD on the testing day (close to 1-g HFD) during the first of 2.5 h, compared with control groups (<0.1 g). This rapid consumption of HFD over a short period of time (2.5 h) in the absence of hunger is analogous to binge-like eating. HFD feeding on the whole testing day (24 h), representing a choice of HFD intake in the presence of both HFD and chow diets, was also recorded to assess 24-h HFD feeding.

As VTA DA neurons have been associated with binge and hedonic eating (Domingos et al., 2011), KO mice were subjected to the intermittent HFD feeding test. Surprisingly, KO mice exhibited significantly higher HFD feeding during the first 2.5-h period (Fig. 9*B*), suggesting an exaggerated binge-like eating. Interestingly, 24-h HFD feeding was significantly reduced in KO mice, compared with controls (Fig. 9*C*), which was associated with a compensatory increased chow feeding (Fig. 9*D*). We also examined the feeding response to leptin in both 2.5- and 24-h feeding. Leptin had no effect on 2.5-h chow (data not shown) or binge-like eating (Fig. 9*E*) in either genotype; or on 24-h chow feeding in either genotype (Fig. 9*G*). However, leptin significantly reduced 24-h hedonic HFD feeding in controls but had no effects in KO mice (Fig. 9*F*).

## Discussion

Despite extensive research, the neural pathway underlying leptin action remains elusive. Our current results showed that, in all brain regions with LepR expression, only those in the midbrain express VMAT2. Using a combination of mouse genetics, electrophysiology, optogenetics and DREADD, our results demonstrate an importance of VMAT2-mediated neurotransmission in mediating the leptin action on HFD feeding and diet-induced obesity.

Stimulation of ChR2-expressing fibers of VTA LepR neurons induced both oEPSCs and oIPSCs in the amygdala, a region that receive abundant VTA LepR neuron projections, and the accumbens, a region that receives relatively less VTA LepR neuron projections (Leshan et al., 2010). oEPSCs recorded in both sites were shown to be mediated by monosynaptic connectivity, which is consistent with the observation that a subset of VTA LepR neurons expressed VGLUT2. Consistently, VMAT2 deletion caused no difference in oEPSC responses evoked by photostimulation of VTA LepR neuron fibers. In amygdalar neurons, IPSCs elicited by photostimulation were delayed, compared with that of oEPSCs, and sensitive to TTX/4-AP, suggesting that the detected oIPSCs were not due to direct GABA release from LepR neurons, but rather reflected local GABAergic circuits. In contrast to the amygdala, both oEPSCs and oIPSCs detected in the accumbens were mediated by monosynaptic connectivity and independent of VMAT2. Previous observations demonstrated that VMAT2 mediates both DA and GABA release (Tritsch et al., 2012; Tritsch et al., 2014; Kim et al., 2015). One possibility underlying the apparent discrepancy is that VMAT2-mediated GABA release only occurs in non-LepR neurons. Supporting this, only 25% of VTA TH neurons express ALDH1a1, an enzyme that is required to synthesize GABA for VMAT2-mediated release (Kim et al., 2015). Another possibility is that VMAT2-mediated GABA release from VTA LepR neurons only accounts for a minor portion of total GABA release, and disrupted GABA release owing to VMAT2 deletion failed to cause a significant change in the evoked GABA release. Of note, VMAT2-independent, monosynaptic IPSCs detected in accumbens neurons are probably mediated by VGAT, which is expressed in a subset of LepR neurons. Thus, these results suggest that VMAT2-mediated GABA release from VTA LepR neurons, if exists, contributes insignificantly to total GABA release from these neurons.

Midbrain DA neurons mediating leptin action at least involves direct and indirect mechanisms. The former is due to a direct leptin action on LepR neurons in the midbrain (Hommel et al., 2006) and the latter involves a projection of LH LepR neurons to midbrain DA neurons (Leinninger et al., 2009). While LepR neurons in the LH show mixed, both excitatory and inhibitory, responses to leptin (Leinninger et al., 2009), those in the VTA show mainly inhibitory response to leptin, as supported by our current data as well as previous observations (Hommel et al., 2006). Hyperphagia, obesity and defective locomotion were observed in mice with deficiency of LepR or its key signaling molecule STAT3 in the LH or VTA on chow diet (Hommel et al., 2006; Leinninger et al., 2009; Leinninger et al., 2011; Fernandes et al., 2015). It is thus surprising that *LIC::Vmat2^fl^°^x/fl^°^x^* mice were grossly normal on chow. This discrepancy may be due to either recruitment of other downstream pathways including orexin neurons by LH LepR neurons (Leinninger et al., 2011) or to adult deletion of LepR in the VTA (Hommel et al., 2006), which eliminates potential developmental compensations. Alternatively, since prominent glutamate and GABA release can be detected from LepR neurons, a role for their release cannot be ruled out. Indeed, recent studies have demonstrated an importance for glutamate and GABA release from VTA DA neurons (Brown et al., 2012; Hnasko et al., 2012; van Zessen et al., 2012; Root et al., 2014; Qi et al., 2016).

Leptin action on reward value of HFD feeding also involves both direct effect on VTA neurons (Hommel et al., 2006; Fernandes et al., 2015) and indirect through LH neurons (Leinninger et al., 2009). Here, we demonstrated that mice with VMAT2 deletion in midbrain LepR neurons resisted DIO and consumed less HFD feeding. Interestingly, with an intermittent HFD feeding paradigm, VMAT2 deletion reduced 24-h HFD feeding. It is important to point out that, when facing a choice between chow and HFD, KO animals consumed less HFD feeding and more chow feeding, compared with controls, suggesting a reduced level of hedonic feeding. As leptin imposes an overall inhibitory action on VTA LepR neurons, resistance to diet-induced obesity by VMAT2 deletion is in line with a general role of leptin-inhibited neurons such as AgRP neurons (Tong et al., 2008). Supporting a role for VTA LepR neurons in hedonic feeding, acute activation of these neurons by DREADD exhibits more prominent effects in promoting HFD feeding.

One striking result is that VMAT2 deletion from LepR neurons led to exaggerated acute 2.5-h HFD feeding in a nonfasting, intermittent HFD access setting. The consumption of a large amount of HFD within a short period of 2.5 h when presented with a choice between chow and HFD has been used previously to mimic binge eating (Czyzyk et al., 2010; Cao et al., 2014). The increased binge-like eating is in contrast to the reduced 24h HFD feeding in the same testing paradigm, suggesting different mechanisms for binge and hedonic feeding. Supporting this, while the 24-h hedonic feeding was inhibited by leptin, the binge eating is not sensitive to leptin in both controls or *LIC::Vmat2^fl^°^x/fl^°^x^* mice, suggesting that the binge eating behavior is not modulated by acute leptin action. Given the defective vesicular accumulation by VMAT2 deletion, the exaggerated binge-like eating behavior is in line with human data that binge patients tend to have reduced level of DA in the brain (Bello and Hajnal, 2010). Supporting this, DA receptor 2 antagonism in the accumbens increases binge-like feeding (Halpern et al., 2013) and activation of DA neurons inhibits binge-like eating (Xu et al., 2016). One possible underlying mechanism for the exaggerated binge-like eating is that, with disrupted DA release from LepR neurons, additional HFD feeding is required to achieve comparable amount of DA release in the accumbens for the same degree of perceived pleasure associated with HFD feeding. A similar mechanism has been suggested for drug abuse (Murray et al., 2014). In line with this, VTA neurons are activated during the intermittent HFD access period (Sahr et al., 2008). Alternatively, the overconsumption of HFD may simply reflect an overresponse to the new presentation of nonchow food, rather than a response to hedonic feeding. additional experiments with HFD replaced by another nonhedonic food will be able to test this possibility.

In summary, our study identified an importance for VMAT2-mediated neurotransmission in mediating hedonic feeding and binge-like eating behavior, providing novel insight on the interactive roles of leptin and DA, two of the most extensively studied brain systems, in obesity development.

## Synthesis

Reviewing Editor: Jeffrey Blaustein, University of Massachusetts Decisions are customarily a result of the Reviewing Editor and the peer reviewers coming together and discussing their recommendations until a consensus is reached. When revisions are invited, a fact-based synthesis statement explaining their decision and outlining what is needed to prepare a revision will be listed below. The following reviewer(s) agreed to reveal their identity: Ralph DiLeone, Jonathan Hommel

There is significant interest in your manuscript. There are, however, some issues that will need to be tended to as follows:

1. Figure 3 shows that dopamine cannot be detected (via antibody) in neurons missing VMAT2, as expected. However, they did not measure “DA in vesicles” in a direct way and the stated conclusions should reflect the limitations of the approach used.

2. The authors record in the amygdala and stated that the presence of fibers is consistent with “preferential” projection of Lepr+ midbrain neurons to the amygdala. However, Figure 6 clearly shows quite prominent projections to the nucleus accumbens which would be in contrast to Leshan et al., 2010. The authors state that this is “relatively less” in the Discussion, but report that nearly ~50% of the recorded neurons show oEPSC responses, indicating significant influence on the NAc region. This issue should be clarified.

3. Are the minimal effects of VMAT2 deletion in females due to differential expression? It was not clear if the immunostaining studies were also completed in female mice as this could explain the results.

4. The effect of VMAT2 removal on binge intake (Fig. 9B) is surprising, as noted by the authors, considering the other data presented. The opposite effect on HFD intake (“hedonic eating”) suggests that the animals could be over-responding to presentation of an alternate food (though not novel), regardless of composition. It could be informative to evaluate a non-hedonic food (e.g. flavored) to test if the same effect is seen. Although not necessary to do this experiment at this time, it should be discussed briefly.

5. In Figure 2, p-STAT3 IHC was performed following ICV delivery of leptin. The observed co-localization could be mediated by a “network effect”. That is, the induction of p-STAT3 may be due to activation of another signaling cascade utilizing STAT3, not necessarily due to LepR activation. Thus, direct delivery of leptin into the VTA should be performed. Although not essential at this time, this potential “network effect” should be mentioned in the text.

6. Figure 7 and its description in the results section are unclear. On line 298, did the authors intend to indicate: “These results suggest that loss of VMAT2-mediated neurotransmission from LepR neurons reduces HFD.... ”

Typos etc.

Line 179, “... through fiber.” This is not clear.

Lines 255-259, three sentences in a row start with “Interestingly,...”

Fig. 5B, “oPSC” should be “oEPSC”

Panel 9G is not noted in figure legend and 9F and 9G are misidentified in the text.
